# Bioinformatics analysis of copper death gene in diabetic immune infiltration

**DOI:** 10.1097/MD.0000000000035241

**Published:** 2023-09-29

**Authors:** Zhimin Lu, Ling Ding, Sen Zhang, Xing Jiang, Qinglu Wang, Ying Luo, Xuewen Tian

**Affiliations:** a Shandong Sport University, Jinan, Shangdong Province, China; b Department of Clinical Laboratory, Zibo Central Hospital, Zibo, China.

**Keywords:** bioinformatics, copper death, diabetes mellitus, immune infiltration

## Abstract

**Background:**

Copper plays an important role in the human body and is potentially related to the development of diabetes. The mechanism of copper death gene regulating immune infiltration in diabetes has not been studied.

**Methods:**

Download microarray data from healthy normal and diabetic patients from the GEO database. The identification of differentially expressed genes (DEGs) was analyzed by gene enrichment. Using String online database and Cytoscape software to interact with the protein interaction network and make visual analysis. Using Wilcox analyze the correlation between the copoer death gene and diabetic mellitus. Analysis of the correlation between immune penetration cells and functions, and the difference between the diabetes group and the control group, screening the copper death gene associated with diabetes, and predicting the upper top of microRNA (miRNA) through the Funrich software.

**Results:**

According to the identification of differential genes in 25 samples of GSE25724 and GSE95849 data sets, 328 differential genes were identified by consensus, including 190 up-regulated genes and 138 down-regulated genes (log2FC = 2, *P* < .01). KEGG results showed that neurodegeneration-multiple disease pathways were most significantly upregulated, followed by Huntington disease. According to Cytohubba, the TOP10 genes *HCK, FPR1, MNDA, AQP9, TLR8, CXCR1, CSF3R, VNN2, TLR4*, and *CCR5* are down-regulated genes, which are mostly enriched in neutrophils. Immunoinfiltration-related heat maps show that Macrophage was strongly positively correlated with Activated dendritic cell, Mast cell, Neutrophil, and Regulatory T cell showed a strong positive correlation. Neutrophil was strongly positively correlated with Activated dendritic cell, Mast cell, and Regulatory T cell. Differential analysis of immune infiltration showed that Neutroph, Mast cell, Activated B cell, Macrophage and Eosinophil were significantly increased in the diabetic group. Central memory CD4 T cell (*P* < .001), Plasmacytoid dendritic cell, Immature dendritic cell, and Central memory CD8 T cell, etal were significantly decreased. *DBT, SLC31A1, ATP7A, LIAS, ATP7B, PDHA1, DLST, PDHB, GCSH, LIPT1, DLD, FDX1*, and *DLAT* genes were significantly associated with one or more cells and their functions in immune invasion. Forty-one miRNA.

**Conclusions:**

Copper death is closely related to the occurrence of diabetes. Copper death genes may play an important role in the immune infiltration of diabetes.

## 1. Introduction

In recent years, with the increasing number of diabetes, the World Health Organization has listed diabetes as 1 of the 4 major non-communicable diseases warrant attention, and the global death caused by diabetes has reached 31.1% as till 2016.^[1]^ According to the International Diabetes Federation in 2019, the global prevalence of diabetes is 9% (463 million adults).^[2]^ Type 2 diabetes (T2DM) is a disease in which blood sugar levels are elevated, mainly due to insulin resistance (IR) or insufficient insulin secretion, and the incidence is increasing worldwide.^[3]^ T2DM can lead to a series of complications, such as coronary heart disease, kidney disease, retinopathy or neurological disease, and diabetes patients have a higher all-cause mortality.^[4]^

As we all know, copper is an essential factor in the human body, as a transition metal element, mostly in the form of copper ions (Cu2+), which plays a crucial role in maintaining human metabolism.^[5]^ Copper is a “double-edged sword,” when too little copper ion in the body, the cell cannot survive, and too high will lead to abnormal cell function, and even cause cell death.^[6]^ When Cu2 + exists, it can produce reactive oxygen species (ROS) through Fenton reaction or Haber-Weiss reaction, and ROS in the body can promote IR and diabetes. At present, the research on the role of nutrients in diabetes mostly focuses on carbohydrates, while ignoring the research on trace elements. Copper, as an essential trace element in the human body, is also very important for the impact of diabetes.^[7]^ Copper-dependent death occurs through direct binding of copper to the lipidized component of the tricarboxylic acid cycle, which leads to lipidized protein aggregation and subsequent loss of iron-sulfur tuftin, resulting in protein-toxic stress and eventual cell death.^[8]^ Copper death is a newly discovered mode of cell death, which is different from scorch death and cell necrosis, and similar to iron death. Abnormal copper metabolism can lead to a variety of diseases in the human body, such as obesity, cardiovascular disease, tumors, and Menkes disease.^[9–11]^

Diabetes can cause imbalance of copper homeostasis in the body.^[12]^ Some studies have pointed out that compared with non-diabetic control group, the serum copper content of T2DM patients is significantly reduced, and the urinary copper excretion is increased,^[13–15]^ and it was positively correlated with ROS generation.^[16]^ The decrease of serum copper means that Cu2+ accumulate after entering some cells of the body. Then, the decreased expression of ATP7A and ATP7B in diabetic cells resulted in the accumulation of intracellular Cu2+, which was more likely to induce copper death. Based on this, the purpose of this study was to analyze the differential expression of copper-death related genes in diabetic and normal populations, explore the potential expression and interaction of copper-death related genes in diabetic cells, and analyze the internal mechanism of copper-death in diabetic patients, as well as the degree of immune cell and functional infiltration.

## 2. Material and methods

**Figure 1.**
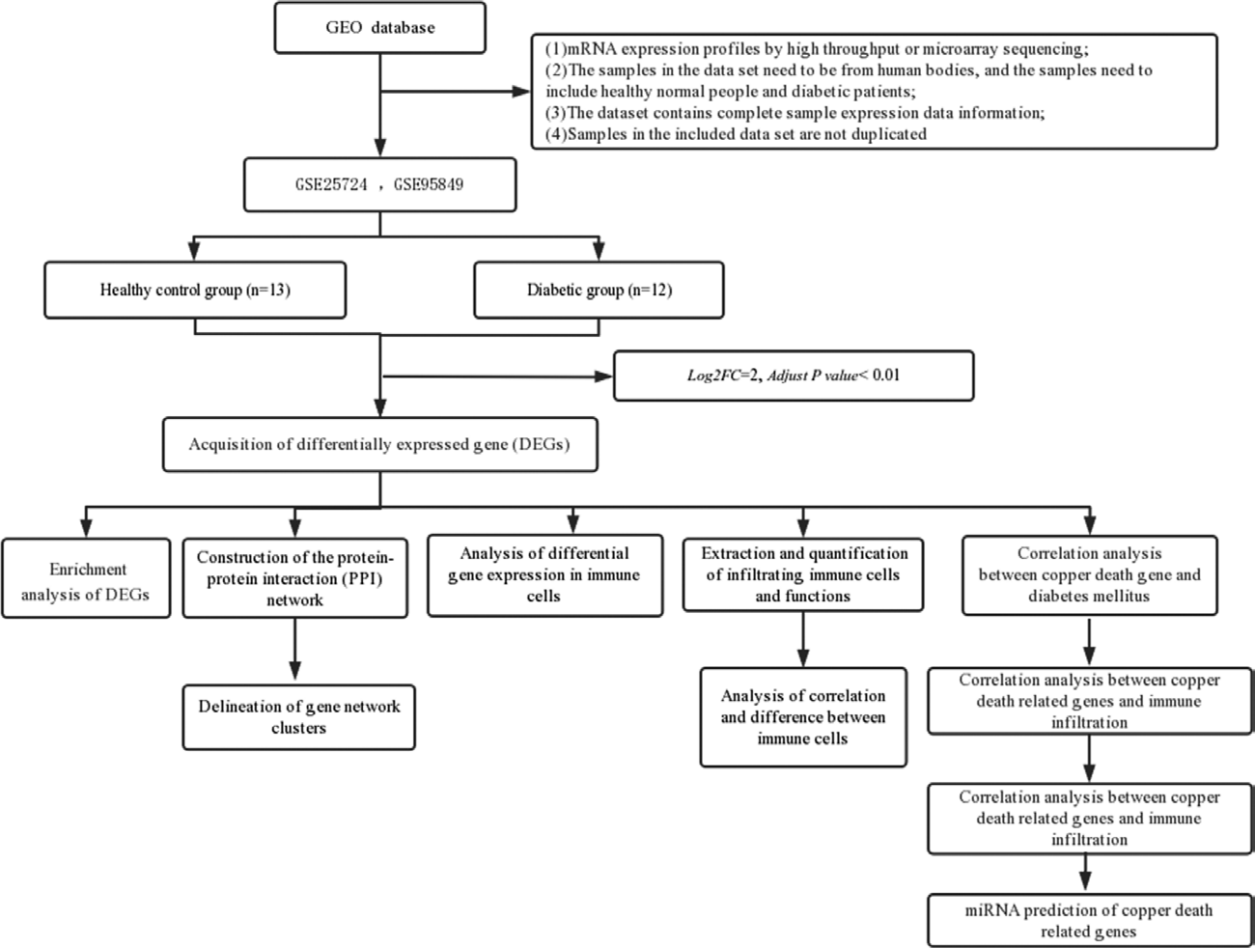
The main process of research and analysis is shown in the figure.

The main process of research and analysis is shown in the Figure 1.

### 2.1. Data source

In order to obtain the microarray gene expression data related to diabetes, this study took “diabetes” as the search term and screened and searched through GEO database. The Ethics committee of Shandong Sport University approved the study.

Inclusion criteria: mRNA expression profiles by high-throughput or microarray sequencing; the samples in the data set need to be from human bodies, and the samples need to include healthy normal people and diabetic patients; the dataset contains complete sample expression data information; and samples in the included data set are not duplicated.

After screening, 2 eligible data sets GSE25724 and GSE95849 were retrieved, with a total of 25 samples, including 13 healthy normal subjects and 12 diabetic patients. The gene data and expression matrix of GSE25724 and GSE95849 were downloaded and analyzed by R software.

### 2.2. Acquisition of differentially expressed genes (DEGs)

Using https://www.aclbi.com/static/index.html#/geo micro needle array average method was carried out on the original file downloaded from GEO database preprocessing and normalization. The lima package was used for genetic analysis of the differences between samples, and multiple hypothesis testing and correction were performed after obtaining *P* values. The *P* value of DEG is determined by controlling the error discovery rate, and the *P* value is adjusted. The threshold *P* value of DEGs is determined by controlling FDR, and the *P* value is adjusted. The screening criteria was log2FoldChange (log2FC) = 2 and the adjust *P* value < 0.01.

### 2.3. Construction of the protein-protein interaction network and Delineation of gene network clusters

In order to better visualization DEG, use https://www.aclbi.com/static/index.html#/geo to create heat maps and volcanic figure. At the same time, use the heat map package to generate a heat map. The variation of Fold and the corrected *P* values were used to map the differential gene expression volcano. The dots in the figure represent genes with significant differences up and down regulated. Forming differential gene expression heat maps, where different colors represent expression trends in different tissues. The protein-protein interaction network for the DEGs we identified was constructed using the STRING database (https://string-db.org) (Stockholm, Swiss), a precomputed database in which associations between proteins are based on high-throughput experiments, gene fusion, co-occurrence, literature mining, co-expression analysis, and computational predictive allocation. Interactions are visualized by Cytoscape v3.9.1 (https://cytoscape.org/). The protein interaction network is constructed using MCODE, and Cytohubba computs the TOP gene.

### 2.4. Analysis of differential gene expression in immune cells

By using the human protein atlas database (https://www.proteinatlas.org/) analysis need differences in gene expression in immune cells.

### 2.5. Enrichment analysis of DEGs

Trends in the distribution of predefined genomes in the gene list are assessed with GSEA to determine their contribution to the phenotype. The GSEA_4.1.0 and c5: The gene ontology (GO) gene sets (C5.all.v7.1.(gmt)) were downloaded for functional enrichment analysis. Then use https://www.aclbi.com/static/index.html#/geo DEG enrichment condition is analyzed, and created a string diagram to visualize the results of these rich. KOBAS 3.0 is a widely used for gene/protein concentration and functional annotation of online database (http://kobas.cbi.pku.edu.cn/kobas3), and is used to DEGs Kyoto encyclopedia of genes and genomes (KEGG) pathway enrichment analysis and response. The significantly enriched pathways and functions were selected using log2FC = 2 and adjust *P* value* <* 0.01.

### 2.6. Extraction and quantification of infiltrating immune cells and functions

In order to extract and quantify the transformed gene expression matrix of infiltrating immune cells, the transformed expression matrix was scored by single-sample gene set enrichment analysis (ssGSEA). ssGSEA is a tool for immunoinfiltration analysis, which contains a total of 29 immune scores to estimate the degree of immunoinfiltration in each sample and to map hotspots using the “pheatmap” software package.

### 2.7. Analysis of correlation and difference between immune cells

In order to analyze the correlation between immune cells and immune functions, based on the results of ssGSEA expression matrix, corrplo software package in R language was used for correlation analysis and heat map was drawn. In addition, in order to compare the differences of immune cells and functions between the diabetic group and the control group, the sample grouping was integrated with the results of ssGSEA expression matrix, and the rank sum test was carried out through the “ggpubr” R language software package, and the box map was drawn.

### 2.8. Correlation analysis between copper death gene and diabetes mellitus

Studies have reported that the main genes related to copper death are *SLC31A1, PDHB, PDHA1, LIPT1, FDX1, DLD, DLST, DBT, LIAS, DLAT, GCSH, ATP7A*, and *ATP7B.*^[8]^This study analyzed the significance of these genes in 2 groups of samples, diabetes and normal people, and made box maps by Wilcox test.

### 2.9. Correlation analysis between copper death related genes and immune infiltration

In order to analyze the correlation of copper death gene in immune cells and function, the 2 result files of ssGSEA and copper death gene expression matrix were analyzed using the “psych” R language software package, and the correlation heat map was constructed.

### 2.10. microRNA (miRNA) prediction of copper death related genes

Functional enrichment analysis tool (FunRich) software is used for protein gene functional enrichment and interaction network analysis. In order to further understand the expression mechanism of copper death related genes, FunRich software was used in this study to predict the upstream miRNA, and finally the miRNA-miRNA relationship was input into the Xiantao academic platform to visualize the regulatory network.

## 3. Results

### 3.1. Differential gene screening for GSE25724 and GSE95849

Through the identification of differential genes in 25 samples of GSE25724 and GSE95849 datasets, including 13 samples of healthy normal subjects and 12 samples of diabetic patients, this study identified 328 differential genes, including 190 up-regulated genes and 138 down-regulated genes. Table 1 shows the top 10 up-regulated and down-regulated genes in terms of expression difference. *IAPP, PCSK1, SCG2, CPE, PFN2, SLC17A6, SCGN, NAE1*, and *SERINC1* were significantly up-regulated, while *MEIS2, S100P, EGR1, CXCR2, TNFRSF10C, MYOM2, STEAP4, STEAP4, PGLYRP1, SLPI, MME*, and *MSRB1* were significantly down-regulated.

**Table 1:** Significant up-regulated and down-regulated genes screened according to Log_2_FC.

Up-regulated expressed genes			Down-regulated expressed genes		
Gene	Log2FC	*P*	Gene	Log2FC	*P*
*IAPP* *PCSK1* *SCG2* *CPE* *PFN2* *SLC17A6* *SCGN* *NAE1* *SERINC1* *MEIS2*	2.493 2.194 2.031 1.907 1.707 1.680 1.674 1.636 1.613 1.611	.000 .001 .005 .002 .000 .007 .001 .005 .005 .003	*S100P* *EGR1* *CXCR2* *TNFRSF10C* *MYOM2* *STEAP4* *PGLYRP1* *SLPI* *MME* *MSRB1*	−2.859 −2.779 −2.353 −2.328 −2.305 −2.032 −2.032 −2.025 −1.993 −1.960	.000 .003 .007 .004 .004 .007 .003 .003 .003 .005

Log2FC = log2FoldChange, *P* = *P* value.

The differential genes of GSE25724 and GSE95849 data sets were analyzed. GSE25724 was analyzed and 152 up-regulated genes and 21 down-regulated genes were found. *PCSK1, IAPP, SCG2, CLGN, SCG3, ALDH1A1, GNAI1, ENPP2, QPCT*, and *CPE* were significantly up-regulated, while *CD74, S100P, PGGHG, ISG15, IRF7, LTB, TCIRG1*, and *CXCL10* were significantly down-regulated. CRISP3 and FOSB. GSE95849 had 174 up-regulated genes and 344 down-regulated genes. Significantly upregated genes were *PEG3, RCL1, ZBTB43, CLDN14, RCC1, EXD2, RPL27A, SLC7A5, CAMKV*, and *DRD5*. Significantly down-regulated genes were *MME, MMP9, PI3, S100P, TNFRSF10C, KRT23, MYOM2, STEAP4, EGR1*, and *CXCR2*.

**Figure 2.**
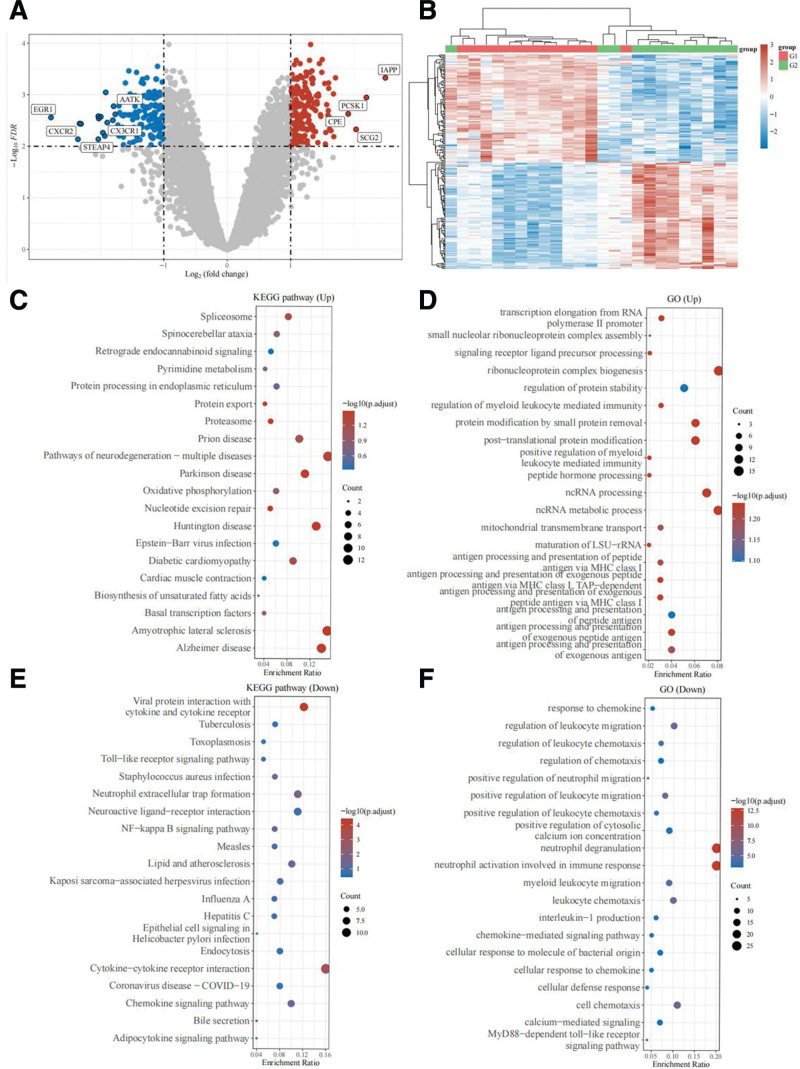
Heat map, volcano map, KEGG, GO analysis results. (A) Differential gene expression volcano map. Red shows significantly up-regulated genes such as *IAPP, PCSK1*, etc. Blue shows significantly up-regulated genes such as *AATK, EGR1*, etc. (B) Differential gene expression heat map. Where different colors represent the expression trend in different organizations. Due to the large number of differenced genes, 50 up-regulated genes and 50 down-regulated genes in the diabetes group were shown here, respectively, compared with the normal group. (C) Up-regulation diagram of signaling pathway. (D) GO analysis up-regulation diagram. (E) Up-regulation diagram of signaling pathway. (F) GO analyze the downdraft. GO = gene ontology, KEGG = Kyoto encyclopedia of genes and genomes.

A volcano map is formed (Fig. 2A), where blue dots represent significantly down-regulated genes, red dots represent significantly up-regulated genes, and gray dots represent genes with no significant differences. The differential gene expression heat map was formed to show the 50 up-regulated genes and 50 down-regulated genes with the largest difference changes respectively (Fig. 2B).

### 3.2. KEGG pathway and GO analyses

KEGG results showed that neurodegeneration -multiple disease pathways were most significantly upregulated, followed by Huntington disease and Parkinson disease (PD) pathways. The cytokine - cytokine receptor interaction pathway was most significantly down-regulated, followed by the viral protein-cytokine and cytokine receptor interaction pathway, and again by the neutrophil extracellular trap formation pathway (Fig. 2C and 2E). GO results showed that the most significant upregulation was in the biogenesis of ribonucleoprotein complex and ncRNA metabolism, followed by ncRNA processing, protein modification by removing small proteins, and post-translational protein modification. Neutrophil activation involved in immune response and neutrophil degranulation were most significantly down-regulated, while other processes were not affected (Fig. 2D and 2F).

### 3.3. Expression of TOP10 genes in immune cells

**Figure 3.**
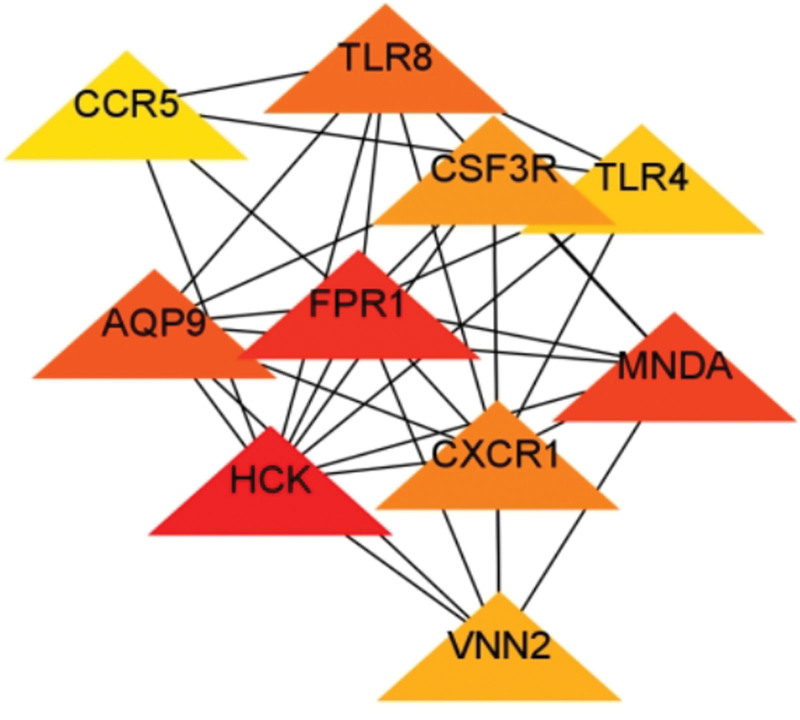
TOP10 PPI network diagram. Using the top10 gene of string online database (https://cn.string-db.org/) and cytosaper software, the construction of the network visualization network. PPI = protein-protein interaction.

**Figure 4.**
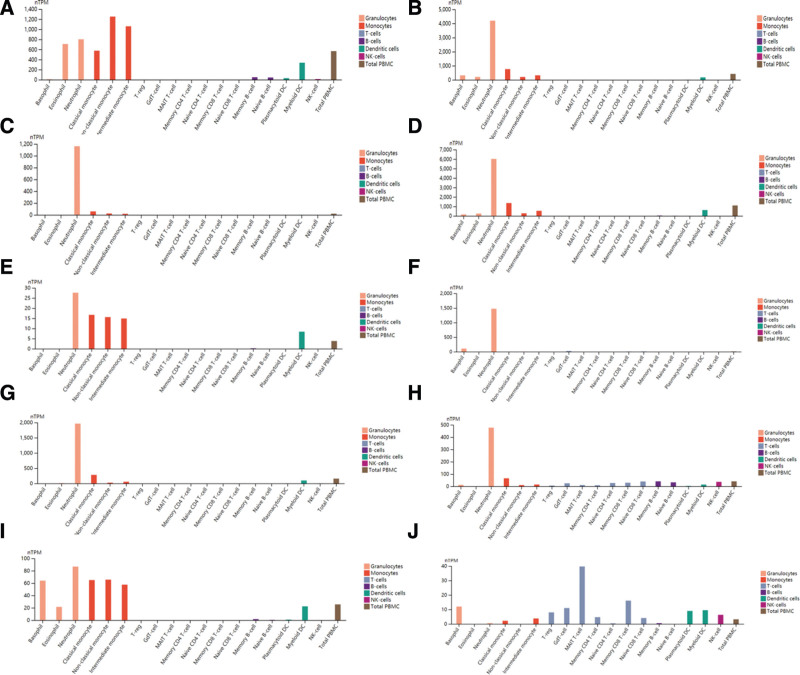
Expression of TOP10 genes in immune cells. (A) *HCK* gene; (B) *FPR1* gene; (C) *MNDA* gene; (D) *AQP9* gene; (E) *TLR8* gene; (F) *CXCR1* gene; (G) *CSF3R* gene; (H) *VNN2* gene; (I) *TLR4* gene; (J) *CCR5* gene.

328 differential genes were analyzed by STRING, and the TOP10 genes were calculated by using Cytoscape plugin MCODE for visual analysis. *HCK, FPR1, MNDA, AQP9, TLR8, CXCR1, CSF3R, VNN2, TLR4*, and *CCR5* (Table 2, Fig. 3), the genes with high scores were down-regulated genes. *HCK* gene was abundant in non-classical monocytes, intermediate monocytes, neutrophils, eosinophils, classical monocytes and myeloid *DC, FPR1, MNDA, AQP9, CXCR1, CSF3R, VNN2*, and *TLR4* genes were enriched in neutrophils. *TLR8* gene was enriched in neutrophils, classical monocytes, non-classical monocytes, intermediate monocytes and myeloid DC. *CCR5* gene was enriched in MAITT cells. It can be seen that the first 10 genes are mostly enriched in neutrophils (Fig. 4).

**Table 2:** TOP10 genes calculated by Cytoscape.

Gene symbol	log2FC	*P*	Regulation	Score
*HCK* *FPR1* *MNDA* *AQP9* *TLR8* *CXCR1* *CSF3R* *VNN2* *TLR4* *CCR5*	−1.489 −1.561 −1.706 −1.723 −1.436 −1.762 −1.237 −1.422 −1.436 −1.499	.003 .002 .006 .004 .002 .005 .001 .007 .002 .001	Down Down Down Down Down Down Down Down Down Down	1777 1663 1262 1128 1052 1023 923 889 865 612

Down = down-regulated, Log2FC = log2FoldChange, *P* = *P* value.

### 3.4. Degree of immune cell and functional infiltration in diabetic patients

**Figure 5.**
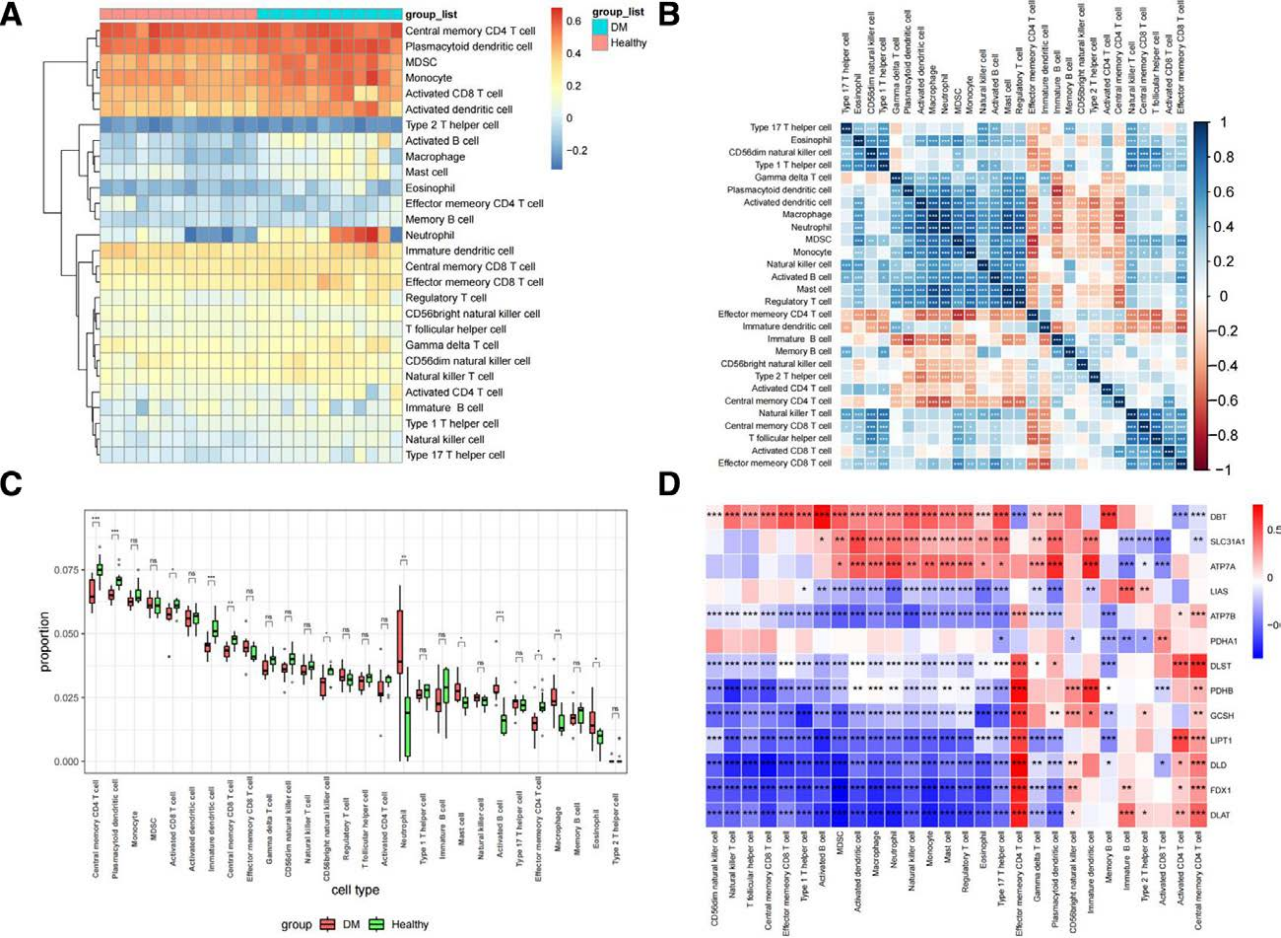
Correlation analysis of immune infiltration. (A) Correlated heat maps of immune infiltration in diabetic and normal samples, the darker the red, the greater the positive correlation, and the darker the blue, the greater the negative correlation. As can be seen from the figure, CD4 cell, Plasmacytoid, etc is significantly positively correlated with the normal group, while Type 2 helper cell, Macrophage, etc is significantly negatively correlated. (B) Heat map of correlation analysis between immune cells. There was a strong positive correlation between cell (*R* = 0.89), Neutrophil (*R* = 0.93) and Regulatory T cell (*R* = 0.82). Neutrophil was strongly positively correlated with Activated dendritic cell (*R* = 0.86), Mast cell (*R* = 0.88), and Regulatory T cell (*R* = 0.86). There was a strong positive correlation between Regulatory T cells and Mast cells (*R* = 0.91). (C) Analysis box of the difference of immune infiltrating cells between the diseased group and the normal group. he results showed that Neutroph (*P <* .01), Mast cell (*P *< .05), Activated B cell (*P *< .001), Macrophage *(P *< .01), and Eosinophil (*P *< .05) were significantly increased in the diabetic group. Central memory CD4 + T cell (*P *< .001), Plasmacytoid dendritic cell (*P *< .001), Immature dendritic cell (*P* < .001), and Central memory CD8 T cell (*P *< .01), CD56 bright natural killer cell (*P* < .05), and Effector memeory CD4 + T cell (*P *< .05) were significantly decreased. (D) Heat map of correlation analysis between copper death gene and immune infiltration. *DBT, SLC31A1, ATP7A, LIAS, ATP7B, PDHA1, DLST, PDHB, GCSH, LIPT1, DLD, FDX1*, and *DLAT* genes were significantly associated with one or more cells and functions in the immune invasion.

ssGSEA was used to analyze 28 immune cells and functional immunity in GSE25724 and GSE95849 datasets. The obtained immune cell-related gene sets included CD4 + T cells, dendritic cells, myeloid suppressor cells, monocytes, CD8 + T cells, dendritic cells, B cells, macrophages, and neutrophils (Fig. 5A).

### 3.5. Correlation and difference analysis of diabetic immune infiltration

The immune cell correlation heat map showed a strong positive correlation between Macrophage and Activated dendritic cell (*R* = 0.83) and Mast There was a strong positive correlation between cell (*R* = 0.89), Neutrophil (*R* = 0.93) and Regulatory T cell (*R* = 0.82). Neutrophil was strongly positively correlated with Activated dendritic cell (*R* = 0.86), Mast cell (*R* = 0.88), and Regulatory T cell (*R* = 0.86). There was a strong positive correlation between Regulatory T cells and Mast cells (*R* = 0.91) (Fig. 5B).

The immunoinfiltrating cells were analyzed between the diabetic patients and the healthy people by the box map, and *P* < .05 showed a significant difference. The results showed that Neutroph (*P *< .01), Mast cell (*P *< .05), Activated B cell (*P *< .001), Macrophage (*P *< .01) and Eosinophil (*P *< .05) were significantly increased in the diabetic group. Central memory CD4 + T cell (*P *< .001), Plasmacytoid dendritic cell (*P *< .001), Immature dendritic cell (*P *< .001), and Central memory CD8 T cell (*P *< .01), CD56 bright natural killer cell (*P *< .05) and Effector memeory CD4 + T cell (*P *< .05) were significantly decreased (Fig. 5C).

### 3.6. Correlation of tissue cell differential gene and copper death gene between diabetic patients and healthy normal subjects

**Figure 6.**
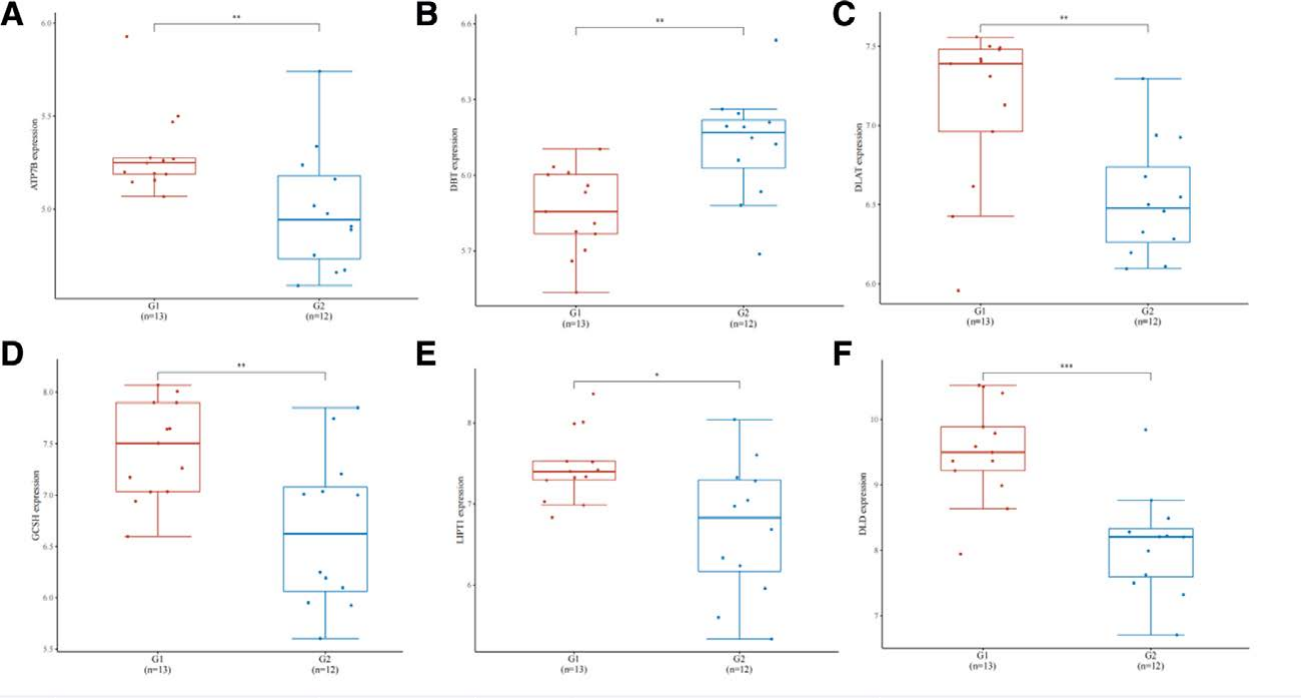
Copper death related genes in tissue cells of diabetic patients and healthy normal subjects.

**Figure 7.**
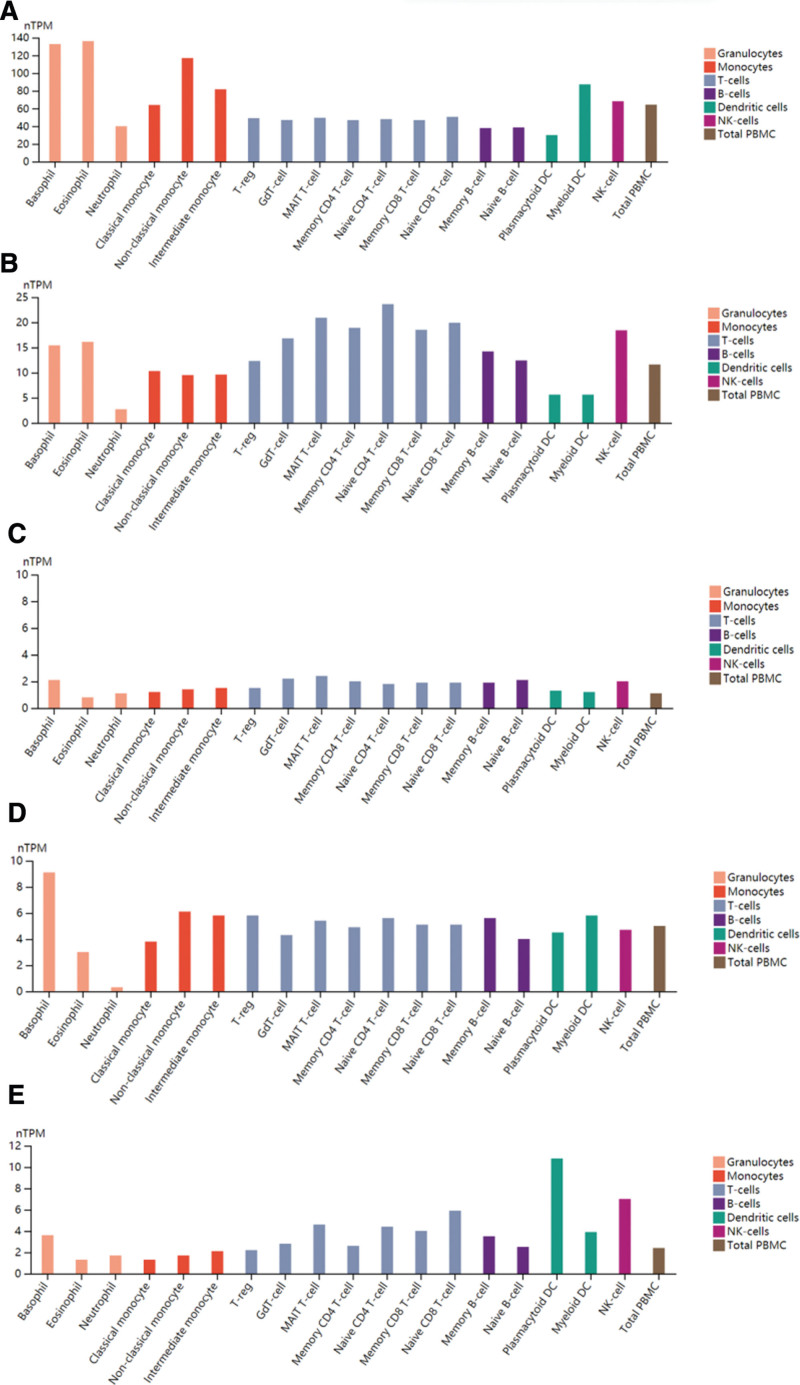
Copper death related genes in diabetic patients and healthy normal tissue cells. The expression levels of *DLD, LIPT1, DBT, DLAT*, and *GCSH* were different in different immune cells.

Compared with healthy controls, the expression of copper death related genes in the tissues of diabetic patients was significantly down-regulated, including *ATP7B (P *= .0096), *DBT (P =* .0019), *DLAT (P *= .003), *GCSH (P =* .0096), and *LIPT1 (P* = .017)*. DLD (P *= .00045) expression was significantly up-regulated (Fig. 6). The results showed that diabetes affected the expression changes of genes in immune cells, especially neutrophils. Based on this, this study further analyzed the expression of genes related to copper death in immune cells, and the expression levels of *DLD, LIPT1, DBT, DLAT*, and *GCSH* were different in different immune cells (Fig. 7).

### 3.7. Correlation analysis between copper death gene and immune infiltration

Based on the 2 result files of ssGSEA and copper death gene expression matrix, the correlation score map of copper death gene and immune cells and function was obtained in this study. The results showed that *DBT, SLC31A1, ATP7A, LIAS, ATP7B, PDHA1, DLST, PDHB, GCSH, LIPT1, DLD, FDX1*, and *DLAT* genes were significantly associated with 1 or more cells and functions in the immune invasion (Fig. 5D).

### 3.8. miRNA analysis of copper death related genes in diabetes mellitus

The upstream miRNA of copper death related genes was predicted using FunRich software. A total of 41 mirnas, including hsa-miR-31-5p, hsa-miR-124-3p, hsa-miR-15a-5p, hsa-miR-16-5p and hsa-miR-148a-3p were obtained (Table 3).

**Table 3:** Upstream miRNA of copper death related genes.

Gene symbol	miRNA
*DBT* *SLC31A1* *ATP7A* *LIAS* *ATP7B* *PDHA1* *DLST* *PDHB* *GCSH* *LIPT1* *DLD* *FDX1* *DLAT*	/ hsa-miR-31-5p; hsa-miR-124-3p hsa-miR-15a-5p; hsa-miR-16-5p; hsa-miR-148a-3p; hsa-miR-223-3p; hsa-miR-15b-5p; hsa-miR-124-3p; hsa-miR-152-3p; hsa-miR-195-5p; hsa-miR-148b-3p; hsa-miR-424-5p; hsa-miR-497-5p; hsa-miR-506-3p; hsa-miR-6838-5p / / hsa-let-7a-5p; hsa-let-7b-5p; hsa-let-7c-5p; hsa-let-7d-5p; hsa-let-7e-5p; hsa-let-7f-5p; hsa-miR-98-5p; hsa-let-7g-5p; hsa-let-7i-5p; hsa-miR-133a-3p; hsa-miR-138-5p; hsa-miR-4458; hsa-miR-4500 hsa-let-7a-5p; hsa-let-7b-5p; hsa-let-7c-5p; hsa-let-7d-5p; hsa-let-7e-5p; hsa-let-7f-5p; hsa-miR-25-3p; hsa-miR-98-5p; hsa-miR-192-5p; hsa-miR-215-5p; hsa-let-7g-5p; hsa-let-7i-5p; hsa-miR-137; hsa-miR-194-5p; hsa-miR-363-3p; hsa-miR-367-3p; hsa-miR-4458; hsa-miR-4500 / / hsa-miR-455-3p / hsa-miR-96-5p; hsa-miR-365a-3p; hsa-miR-365b-3p; hsa-miR-376a-3p; hsa-miR-376b-3p; hsa-miR-1271-5p

miRNA = microRNA.

## 4. Discussion

The number of people with diabetes is increasing rapidly in the world, and copper death is an emerging cell death mode, which is closely related to the occurrence of diabetes. In this study, we analysis the differential genes of diabetic patients compared with healthy controls. Next, the enrichment analysis of differential genes KEGG and GO was performed. Then we analysis the expression of copper death genes in the 2 groups of people, and immunoinfiltration analysis was performed.

Future projections indicate that the absolute number of people with diabetes will increase by 46% by 2045.^[2]^ As an important chronic disease threatening human health, diabetes has become one of the top ten causes of adult death.^[17]^ Diabetes is characterized by hyperglycemia, typical symptoms are “three more and one less” (polydipsia, polydipsia, polyuria, weight loss), and its complications are an important cause of death.^[18]^ More seriously, diabetes is associated with long-term irreversible dysfunction and failure of different organs and tissues such as the kidneys, eyes, heart, blood vessels and nerves.^[19–21]^ Studies have shown that saturated fatty acids can promote apoptosis by inducing ROS and endoplasmic reticulum stress pathways,^[22]^ a similar effect was observed in diabetes.^[23]^

First, we analysis the DEGs between diabetic and healthy samples. In this study, it was found that in diabetic patients, the genes significantly down-regulated were *IAPP, PCSK1, SCG2, CPE, PFN2, SLC17A6, SCGN, NAE1*, and *SERINC1*, while the genes significantly down-regulated were *MEIS2, S100P, EGR1, CXCR2, TNFRSF10C, MYOM2, STEAP4, STEAP4, PGLYRP1, SLPI, MME*, and *MSRB1.* Among them, *IAPP* is involved in apoptosis and signal transduction, and its mediated β cell damage may lead to T2DM islet inflammation and dedifferentiation.^[24]^ SCG2 can be used as a key molecule in memory regulation, and FOS can activate SCG2 gene to form a coordinated neural circuit.^[25]^ *EGR1* can regulate the expression of acetylcholinesterase and participate in the change of cholinergic activity of key brain regions in the course of Alzheimer disease (AD), and play an important role in the special pattern of the occurrence and development of cognitive dysfunction in AD. At the same time, this study found that the signaling pathway that was most significantly upregulated was neurodegenerative- a pathway of multiple diseases, followed by Huntington disease, and then the PD pathway. Neurodegenerative diseases (ND) are a general term for diseases caused by the loss of original structure and function of neurons, among which AD, PD, and dementia are more common ND. Evidence suggests a strong association between T2DM, PD, and copper death,^[26–28]^ both are characterized by abnormal protein accumulation, lysosomal and mitochondrial dysfunction, and chronic systemic inflammation. And studies have shown that ND or complications are associated with copper excess.^[29]^ PD and diabetes have common pathogenesis in the aspects of inflammation, mitochondrial function and oxidative stress. IR is a hallmark of T2DM and may also be an important factor leading to PD. In T2DM, IAPP accumulates in pancreatic cells to form amyloid plaques. Similarly, α-synuclein accumulates in neurons of PD patients. It has been shown that aggregation of α-synuclein in PD occurs faster in the presence of IAPP.^[30]^ In addition, the increase in blood sugar causes the loss of dopaminergic neurons, which increases the risk of diabetes patients developing PD.^[31]^ It can be seen that diabetes is closely related to the occurrence of various neurological diseases.

Next,we analysis the TOP 10 genes, and its expression in immune cells was analyzed. In the study found that the TOP10 genes of diabetic patients were *HCK, FPR1, MNDA, AQP9, TLR8, CXCR1, CSF3R, VNN2, TLR4*, and *CCR5*, which were mainly concentrated in neutrophils. At the same time, the significantly down-regulated gene *CXCR2* is an important GPCR-class drug target, regulates the cellular immune function dominated by neutrophils, and is an important inflammatory regulatory target.^[32]^ In addition, the study found that the TOP10 genes of diabetic patients were *HCK, FPR1, MNDA, AQP9, TLR8, CXCR1, CSF3R, VNN2, TLR4*, and *CCR5*, which were mainly concentrated in neutrophils. At the same time, the significantly down-regulated gene CXCR2 is an important GPCR-class drug target, regulates the cellular immune function dominated by neutrophils, and is an important inflammatory regulatory target.

Then, the difference analysis results of immune infiltration and the results of copper death immune infiltration were analyzed. In addition, combined with the difference analysis results of immune infiltration and the correlation results of copper death immune infiltration, it is not difficult to find that CD4 + T cells, dendritic cells, myeloid suppressor cells, monocytes, CD8 + T cells, B cells, macrophages and eosinophilic cells may play an important role in the regulation of diabetes mellitus by copper death gene. CD4 + T cells can directly or indirectly aggravate IR by secreting IL-17, IFN-γ and other cytokines, which is an important mechanism of IR development and islet β cell damage in T2DM patients.^[33,34]^ Dendritic cells are a group of antigen-presenting cells that secrete immune mediators, cytokines, and chemokines associated with chronic inflammatory diseases.^[35]^ Dendritic cells aggregate in the subcutaneous fat of patients with high fat diet and obesity, and induce pro-inflammatory microenvironment by mediating the production of interleukin-6 (IL-6) by macrophages. IL-6 is an independent risk factor for T2DM. Besides stimulating the increase of acute phase proteins such as C-RP and highly sensitive C-reactive protein in circulation, it also reduces the expression of Glu T4 on cell surface. Increase the overexpression of cytokine transduction inhibition 3, reduce the phosphorylation of IRS and IRS-1 related to phosphatidylinositol 3 kinase signaling pathway stimulated by Ins, inhibit Ins signaling, and cause dysfunction of Ins action. Mast cells are important mediators of obesity, IR and T2DM induced by high fat diet. They can mediate macrophages to infiltrate tissues, secrete pro-inflammatory factors and regulate interferon γ and IL-6.^[36]^ In the chronic inflammatory state, proinflammatory monocytes are recruited in adipose tissue and differentiate into M1 type macrophage phenotypes, and their accumulation leads to the imbalance between M1 and M2 type macrophages, which leads to the development of IR, and promotes the occurrence and development of T2DM and its complications^[37]^ B lymphocytes can affect Th17 proliferation and proinflammatory factor production in T2DM patients, and aggravate chronic low-grade inflammation in T2DM patients.^[38]^ All kinds of immune cells play an important role in the immune infiltration of copper death.

And we analysis the expression of copper death gene between healthy people and diabetic samples. In this study, compared with healthy subjects, copper death genes *ATP7B, DLAT, GCSH, LIPT1*, and *DLD* genes were significantly down-regulated, and DBT genes were up-regulated. At the same time, this study selected the copper death genes *DBT, SLC31A1, ATP7A, LIAS, ATP7B, PDHA1, DLST, PDHB, GCSH, LIPT1, DLD, FDX1*, and *DLAT* associated with diabetic immune invasion. Copper death related genes *DLD, DBT, DLAT, LIPT1*, and *GCSH*, which have low immune cell specificity, are expressed in granulocytes, monocytes, T cells, B cells, dendritic cells, and NK cells, while *ATP7B* is not expressed in immune cells. *LIPT1* also has low specificity in immune cells and is expressed in all immune cells. *DBT* encodes transacylase subunit This gene mutation causes type 2 maple diabetes, plays a metabolic role mainly in the kidney and liver, and has low immune cell specificity and is expressed in all immune cells. *ATP7B* is expressed in liver and CD105 + endothelial cells, and participates in copper ion transport and copper homeostasis in vivo. It can be seen that the occurrence of copper death is potentially related to diabetes.

Next, the function of neutrophils was analyzed. Neutrophils play an important role within the body, exerting their immunomodulatory functions by producing ROS and neutrophil extracellular traps (NETs). When inflammation occurs in the body, neutrophils often migrate to the inflammation site first through the blood circulation, and play an important role in innate immunity, adaptive immunity and functional defense by phagocytosis and degranulation, producing ROS and releasing neutrophils extracellular trapping NETs.^[39,40]^ Under normal physiological conditions, ROS in the body maintains a dynamic balance. However, when acute inflammation occurs in the body, the balance between ROS and antioxidants is broken, and the ROS consumption in the body is reduced, resulting in excess ROS present in the body. It can be seen that the balance of neutrophils is crucial to maintain the normal regulatory function of the body.

At the same time, we analyzed the important role of copper ion in diabetes. The balance of Cu2+ is very important for maintaining homeostasis in human body. When the balance of copper metabolism is unbalanced in the body, cytotoxicity can be generated and copper death process can be induced. Copper death is potentially associated with the development of diabetes. The homeostasis imbalance of trace elements in human body is one of the mechanisms leading to diabetes and its complications.^[41–43]^ One of the pathogenesis of diabetes and diabetes complications is abnormal homeostasis of trace elements.^[44,45]^ At the same time, copper as a trace element plays a vital role in oxidation-reduction reactions, contributing to oxidative stress in excess.^[46,47]^ It can be seen that the copper ion balance in the body plays an important role in the maintenance of body homeostasis, and is closely related to the occurrence of diabetes mellitus.

And the important relationship between neutrophils and copper death was analyzed. The increase and decrease of copper content may lead to the imbalance of the antioxidant system in the body, which directly affects the onset and development of diabetes.^[48]^ Other studies have found that Cu2+ and ROS are involved in the development of T2DM.^[12]^ It can be seen that the occurrence of copper death is closely related to the level of neutrophils in the body.

Finally, we comprehensively analyzed the relationship between copper death, neutrophils and diabetes. Copper deficiency can cause neutropenia in the body,^[49]^ thus disrupting the ROS balance in the body. Copper death is closely related to ROS production in diabetic patients. High blood sugar can upset the balance of the number of neutrophils in the body. Hyperglycemia can induce vascular dysfunction in the presence of neutrophils, leading to body injury.^[50]^ Neutrophil to lymphocyte ratio is the ratio of the absolute value of neutrophils to the absolute value of lymphocytes. Neutrophils were positively associated with urinary albumin secretion in T2DM and negatively associated with lymphocytes.^[51]^ Studies have shown that neutrophil to lymphocyte values are closely related to the occurrence of diabetic nephropathy and increase the risk of diabetic nephropathy progression. In diabetes, neutrophils are abnormally activated to produce more superoxides and cytokines such as tumor necrosis factor-α, IL-6, and ROS,^[52–55]^ it promoted the production of neutrophil NETs.^[56,57]^ Most of the destructive effects of superoxides are caused by the formation of copper-dependent ROS.^[12]^ Tanaka, A and Kaur, B et al found that ROS levels were elevated in diabetic mice.^[58]^ Masad, A et al found that free Cu2 + can promote ROS production, which is related to T2DM.^[16]^ Galhardi, C.M., et al, have shown that elevated copper intake can cause renal dysfunction and oxidative stress.^[46]^ In T2DM patients, Cu2 + does not protect against lipid peroxidation and ROS.^[47]^ In addition, excess copper may displace enzymatically promoted zinc, thereby inducing oxidative stress in diabetic patients.^[59,60]^ It can be seen that copper death and neutrophils play a crucial role in the human body and affect the occurrence and development of diabetes.

In addition, this study also used FunRich software to predict the upstream mirnas of copper death related genes, and a total of 41 related mirnas were obtained. With the increase of the amount of biosequencing data, the prediction of the association between non-coding RNA and disease through bioinformatics analysis is very beneficial to the further development of subsequent biological experiments. Predicting and quantifying the association between human non-coding RNA and disease through big data analysis can effectively find the most relevant diseases for experimental verification, thereby reducing the time and cost of biological experiments.

## 5. Conclusion

In summary, copper death is closely related to the occurrence of diabetes and its complications of ND such as PD and Huntington disease. Diabetes may change the copper death-related genes in neutrophils to induce ROS promoting complications. The mechanism may be that excessive glucose in blood induces the expression changes of copper death-related genes in neutrophils, and changes the ROS release and NETs function of neutrophils, thus aggravating complications such as ND. In this study, we analyzed not only the correlation between immune infiltration and their differences in diabetes and control groups, but also the correlation between copper death genes and immune cells and functions through the use of diabetes-related microchips and bioinformatics technology. It has certain reference value for further research on the immunity of diabetics and the correlation of copper death mechanism in this field.

However, there are still some limitations of this study. Although there are few studies on the mechanism of copper death related genes in the immune regulation of diabetes, according to the previous studies and the results of this study, it can be inferred that copper death genes may play an important role in the immune infiltration of diabetes, and how to regulate the copper homeostasis of immune cells to prevent the occurrence of diabetes needs to be further studied.

This study can provide some help for the treatment of diabetic patients, and provides a feasible direction for future research on the mechanism of copper death and diabetes.

## Acknowledgments

We thank LetPub (www.letpub.com) for its linguistic assistance during the preparation of this manuscript.

## Author contributions

**Conceptualization:** Zhimin Lu, Xuewen Tian.

**Data curation:** Ling Ding, Sen Zhang.

**Investigation:** Xing Jiang.

**Visualization:** Zhimin Lu.

**Writing – original draft:** Zhimin Lu.

**Writing – review & editing:** Qinglu Wang, Ying Luo, Xuewen Tian.
